# Quantum Dot Nanobead-Based Fluorescence-Linked Immunosorbent Assay for Detection of Glycinin in Soybeans and Soy Products

**DOI:** 10.3390/molecules27123664

**Published:** 2022-06-07

**Authors:** Qinglong Song, Anguo Liu, Shimin Zhang, Runxian Li, Shiyan Qiao, Pingli He

**Affiliations:** State Key Laboratory of Animal Nutrition, College of Animal Science and Technology, China Agricultural University, Beijing 100193, China; sql19972002@126.com (Q.S.); angelo524@foxmail.com (A.L.); zhangshimin@cau.edu.cn (S.Z.); lrx1024@cau.edu.cn (R.L.); qiaoshiyan@cau.edu.cn (S.Q.)

**Keywords:** glycinin, quantum dot nanobead, fluorescence-linked immunosorbent assay, soybeans, soy products

## Abstract

Soybean glycinin, as a major soybean allergen, is difficult to accurately quantify due to its large molecular weight and complex structure. CdSe/ZnS quantum dot nanobead (QB) is a core/shell fluorescent nanomaterial with strong fluorescent signals and high sensitivity at 630 nm. An immunosorbent assay based on CdSe/ZnS quantum dot nanobeads (QBs-FLISA) was developed for the glycinin quantification in soybean and soybean products. Here, the purified glycinin was coated on the microporous plate to serve as the coating antigen, and CdSe/ZnS nanobead conjugated with anti-glycinin polyclonal antibodies was used as fluorescent detection probe. The target glycinin in the sample and the coated antigen on the plate competitively adsorbed the antibody labeled the CdSe/ZnS QBs probes. The limits of detection and quantitation for glycinin were 0.035 and 0.078 μg mL^−1^, respectively. The recoveries of the spiked samples ranged from 89.8% to 105.6%, with relative standard deviation less than 8.6%. However, compared with ELISA, the sensitivities of QBs-FLISA for the detection of glycinin were increased by 7 times, and the detection time was shortened by two-thirds. This QBs-FLISA method has been effectively applied to the detection of soybean seeds with different varieties and soy products with different processing techniques, which will provide a rapid screening method for soybean and soybean products with low allergens.

## 1. Introduction

Soybean is a significant plant protein source widely employed in the food and feed industries because of its high crude protein content [1]. However, some proteins in soybean, such as glycinin and β-conglycinin, have been identified as allergens for humans and animals. By interfering with the stimulating adverse physiological responses and altering normal metabolism, these allergens threaten human and animal health especially at a young age reducing their efficiency in food and animal production [2].

Glycinin (11S), also called Gly m 6, is the primary storage protein in soybean, accounting for 19.5–23.1% of the total protein in soybean and about 40% of the total globulins [2,3]. The molecular mass of glycinin is 300–380 kDa [4]. Glycinin has been found as a significant allergen in feeds. When soy protein-containing diets are fed to young animals, the majority of the glycinin is degraded into peptides and amino acids, and only about 0.002% of glycinin passes through the intercellular or intercellular spaces of the small intestinal epithelial cells into the blood and lymph, stimulating the intestinal immune tissues and producing delayed allergic reactions mediated by T lymphocytes, and ultimately leading to diarrhea [5]. As a result, soybean breeders are developing new cultivars with low glycinin content by selective breeding or other gene-targeting technologies. Researchers also minimize anti-nutritional activity of glycinin through a range of techniques, including expansion, fermentation, and enzymatic hydrolysis [6,7,8,9].

The isolation and purification of glycinin from soybean is a global concern. Over the past decades, considerable efforts have been focused on developing powerful techniques for the isolation of glycinin, such as isoelectric precipitation [10], salt fractionation [11], affinity chromatography [12], pepsin decomposition [13], and phytic acid enzyme degradation [14]. However, the purity of glycinin obtained using these procedures is insufficient for quantitative applications. Quantitative detection methods for glycinin mainly rely on electrophoresis, antiserum, and high-performance liquid chromatography analysis. Gel electrophoresis was the first method used to analyze glycinin [15]. However, gel electrophoretic resolution depends upon the molecular weight distribution of proteins, and several subunit bands of glycinin overlap greatly with other protein subunits of soybean. For example, the acidic and basic subunit’s molecular mass are 34 to 44 kDa and 20 kDa, which overlap with the β subunit of β-conglycinin and trypsin inhibitor factor, respectively. The application of liquid chromatography and liquid chromatography-mass spectrometry (HPLC-MS/MS) techniques for soybean protein detection is relatively new. Mujoo et al. researched a method for detecting glycinin in soybeans by combining reversed-phase high performance liquid chromatography (RP-HPLC) with sodium dodecyl sulfate-polyacrylamide gel electrophoresis (SDS-PAGE) [3]. Houston et al. and Hill et al. used the protein absolute quantification (AQUA) technology with HPLC-MS/MS for the quantification of five subunits of glycinin [16,17]. These methods require long pretreatment time and expensive equipment. Moreover, liquid chromatography could not detect protein activity, while active glycinin is the main cause of allergies in humans and animals.

Previously, enzyme-linked immunosorbent assay (ELISA) was one of the most widely commercialized glycinin fast screening methods because of its good practicability and sensitivity. Ma et al. developed a competitive ELISA utilizing Mab 4B2 for the detection of glycinin [18]. However, this assay took several hours for antibody incubation and enzyme processing. Recently, fluorescence-linked immunosorbent assay (FLISA) has been an effective alternative method since the signal labels are fluorescent materials with high sensitivity. In particular, quantum dot nanobead (QB) is a polymer nanobead combined with a large number of quantum dots, it is thousands of times brighter than a single quantum dot. So, the detection sensitivity can be enhanced [19,20,21]. Compared with fluorescent organic dyes, QB has outstanding optical performance, such as strong luminous intensity, high optical stability, adjustable emission, and narrow photoluminescence spectra [22,23,24]. The surface of the QB is modified with functional groups (carboxyl groups), which can efficiently be directly covalently bound to antibodies and detected using fluorescence spectrophotometer. Here, CdSe/ZnS QB was coupled with anti-glycinin polyclonal antibodies (pAbs) to be used both as target binding probe and detectable signal to prepare an immunosorbent assay (QBs-FLISA). The QBs-FLISA has been employed in the determination of glycinin in soybeans and soy products with enhanced sensitivity and short detection time. To our knowledge, it is the first time that QB has been applied to the FLISA detection of soybean glycinin, which also provides a versatile platform for the detection of other allergens.

## 2. Results and Discussion

### 2.1. The Working Principle of QB_S_-FLISA

**Figure 1:**
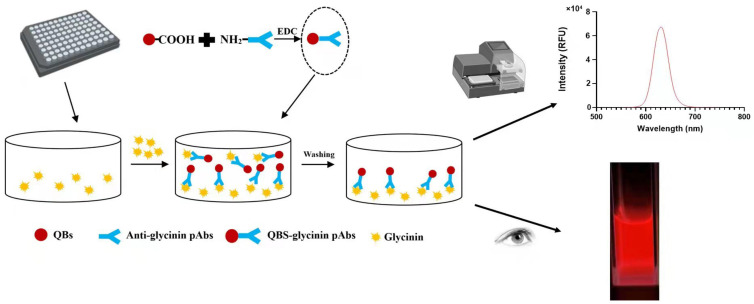
The working principle of QBs-based FLISA for glycinin detection.

Figure 1 illustrates the QBs-FLISA procedure for the detection of glycinin. Firstly, the purified glycinin is coated on the microplate to serve as the capture antigen. Then the sample extractions (or glycinin standard solution) and QB-pAbs probes are added to the wells synchronously. Then, coating antigen and target glycinin in solution competitively associate with QB-pAbs probes. If there is low glycinin concentration in solution, QB-pAbs will bind primarily to glycinin coated on the plate, which exhibits strong fluorescent signals. Conversely, if there is high glycinin in solution, only a small amount of QB-pAbs will bind to glycinin coated on the plate, for which the fluorescent signals will weaken. The fluorescence intensities of QB-pAbs probes adsorbed on the plate are opposite to glycinin concentrations in the samples, which can realize the quantification of glycinin using the calibration curve of standard solutions.

### 2.2. Identification of QB and QB-pAbs Probes

**Figure 2:**
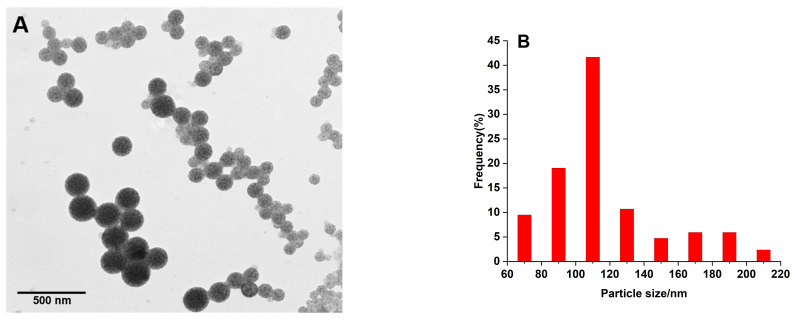

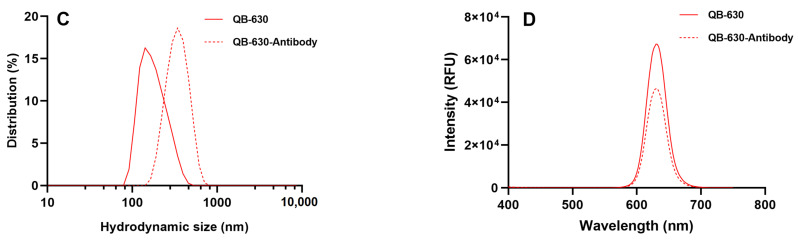

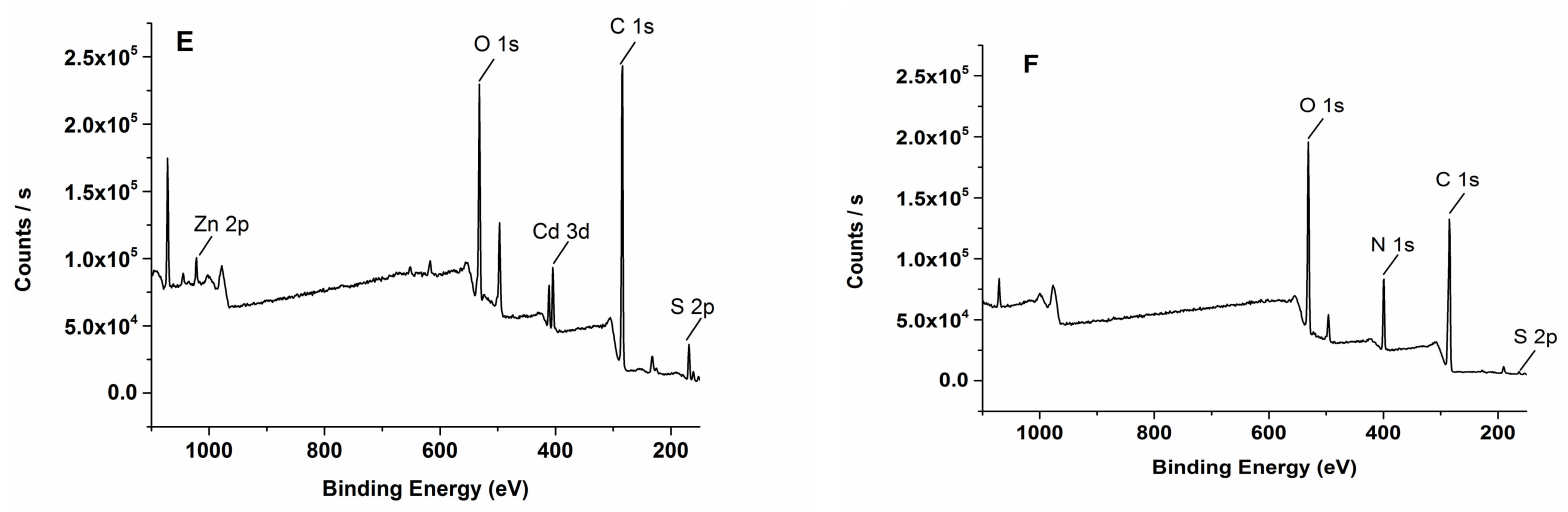
Characterization of QB and QB-pAbs. (**A**) TEM picture of QB. (**B**) Size distribution of QB obtained by TEM. (**C**) Size distribution of QB (solid line) and QB-pAbs probes (dash line) obtained by DLS. (**D**) Fluorescent emission spectra of QB (solid line) and QB-pAbs probes (dash line). (**E**) XPS spectra of QB. (**F**) XPS spectra of QB-pAbs probes.

The QB was synthesized by a self-assembly process of CdSe/ZnS quantum dots embedding into polyethylene nanobeads. The morphology of QB was shown in Figure 2A, TEM picture demonstrates that the nanobeads were uniform and monodispersed, and a large number of quantum dots could be distinctly observed in each QB polymer matrix. According to the magnification scale of the TEM image, we manually measured the diameter range of the main particles in the image. They mainly ranged from 68 nm to 210 nm, with an average of 117 ± 33 nm. We also investigated the dynamic light scattering (DLS) of QB; it had an average hydrodynamic diameter of 185.3 ± 67.5 nm, which was slightly bigger than the diameter determined by TEM (Figure 2B). After conjugation with anti-glycinin pAbs, QB rose in the average hydrodynamic diameter from 185.3 to 351.9 nm (Figure 2C). Therefore, the QB-pAbs conjugates had been effectively synthesized. The highest emission wavelength of QB was 630 nm when excited at 305 nm (Figure 2D). There was no peak shift in QB-pAbs compared with QB, but the fluorescent intensity of QB-pAbs decreased. The centrifugal process may result in the loss of quantum dots. To further analyze whether the quantum dots and antibodies were successfully crosslinked, X-ray photoelectron spectroscopy (XPS) was used to detect the major elements in the QB and QB-pAbs conjugates. The results show that there was a strong peak of nitrogen at 406 eV for QB-pAbs conjugates; however, the QBs did not show this peak (Figure 2E, F). Since QB is composed of CeSe/Zns and polyethylene, there was no nitrogen element. The nitrogen element of QB-pAbs was from antibody protein. The results proved that the antibodies have successfully crosslinked to the quantum dot microspheres.

### 2.3. Optimization of Sample Pretreatment

**Figure 3:**
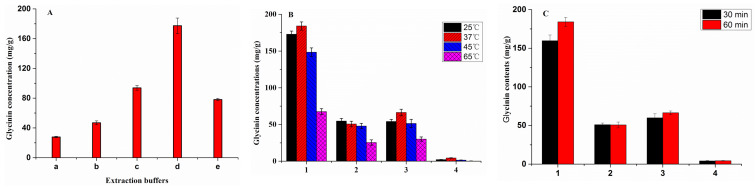
(**A**) Optimization of methods for extraction buffer solutions on glycinin extraction efficiency of soybean meals (*n* = 3). (a) 0.01 M PBS (pH 8.0); (b) 50 mM Tris-HCl (pH 9.0); (c) 50 mM Tris-HCl (pH 10.0); (d) 50 mM Tris-HCl buffer (pH 10.0, 0.9% β-mercaptoethanol); (e) Aqueous solution of sodium hydroxide (pH 10.0). (**B**) Optimization of methods for 1 h extraction temperature of glycinin (*n* = 3). (1) Soybean meals; (2) extruded full-fat soybean; (3) Fermented soybean meal; (4) proteolytic soybean meal. (**C**) Optimization of the extraction time on glycinin extraction efficiency of soybean products (*n* = 3). (1) Soybean meals; (2) extruded full-fat soybean; (3) fermented soybean meal; (4) proteolytic soybean meal.

There are three major factors that can affect the extraction efficiency in soybean and soy products: extraction solvent, extraction time, and extraction temperature. Five extracts, including 0.01 M PBS (pH 8.0), 50 mM Tris-HCl (pH 9.0), 50 mM Tris-HCl (pH 10.0), 50 mM Tris–HCl buffer (0.9% β-mercaptoethanol, pH 10.0), and aqueous solution of sodium hydroxide (pH 10.0), were compared in order to acquire the optimal glycinin extraction efficiency. The results show that the glycinin content in 50 mM Tris-HCl buffer solutions (pH 10.0, 0.9% β-mercaptoethanol) extract was the highest (Figure 3A). Since the isoelectric point of glycinin is 6.4, it is easier to dissolve under alkaline conditions. Furthermore, due to the presence of numerous sulfur-containing amino acid residues in glycinin, adding an appropriate amount of mercaptoethanol can disrupt glycinin disulfide bonds, alter the spatial structure, and enhance solubility. Additionally, the heating temperature was optimized. The results show that shaking resulted in good extraction with vortex oscillation (260 rpm) at 37 °C for 1 h (Figure 3B,C).

### 2.4. Assay Validation of QBs-FLISA

**Figure 4:**
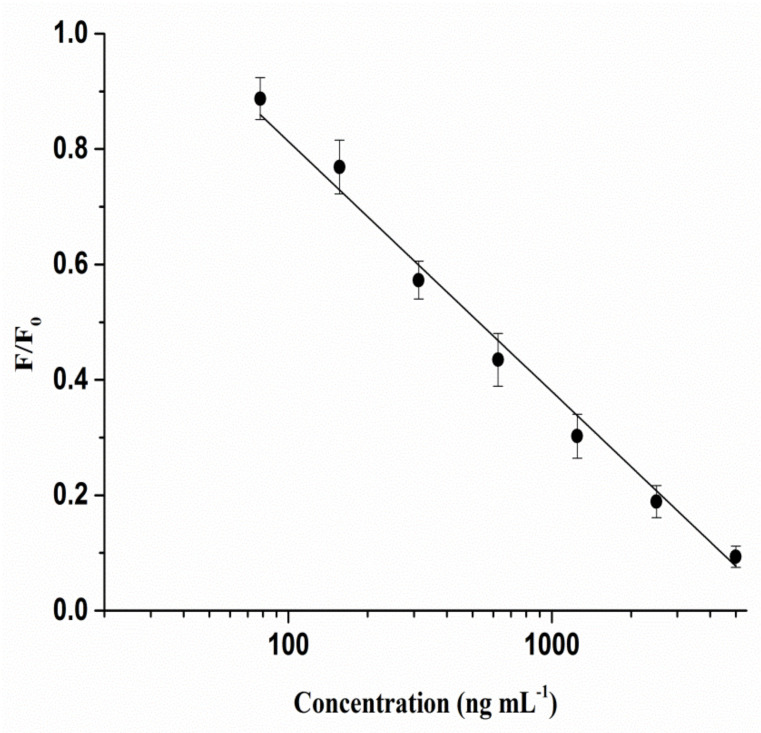
The calibration curve of QBs-FLISA for glycinin.

**Figure 5:**
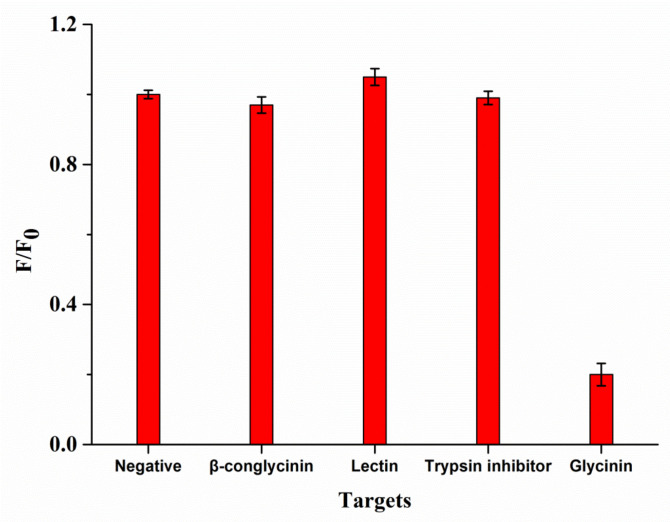
Specificity of QBs-FLISA. The concentration of glycinin is 0.62 μg mL^−1^. The concentrations of β-conglycinin, trypsin inhibitor, and lectin were 20 μg mL^−1^.

Before validation, coating concentrations of glycinin and QB-pAbs dilution ratios were optimized using checkerboard to obtain the optimal analytical performance of QBs-FLISA. As listed in Table 1, the fluorescent intensity decreased with the concentrations decrease of coated antigens and QB-pAbs. The fluorescent intensity was optimal when coating concentrations of glycinin were 5 μg mL^−1^ and QB-pAbs dilution ratios was 1:40. Under optimal assembly conditions, linearity, sensitivity, accuracy, precision, and specificity of QBs-FLISA were evaluated. Firstly, the calibration curve was plotted with the logarithm of the glycinin concentrations as the abscissa and F/F0 as the ordinate. The calibration curve was presented as the mean ± SE (standard error). As shown in Figure 4, the linear range of calibration curve ranged from 0.078 to 5 µg mL^−1^, and the linear fit equation of glycinin was Y = −0.433X + 1.679 (R^2^ = 0.99). The LOD and LOQ were 0.035 μg mL^−1^ and 0.078 μg mL^−1^, respectively. Secondly, accuracy evaluation of the established QBs-FLISA was conducted by determining the recoveries of the samples spiked with purified glycinin solution in different soybean products with three concentrations (40, 80, and 120 mg g^−1^). The average recoveries are shown in Table 2. Recoveries of glycinin in different samples ranged from 89.8% to 105.6%, which showed that this method had favorable accuracy for the quantitative determination of glycinin. The RSD of 6 replications were all less than 8.6%, indicating that the QBs-FLISA was favorably repeatable. Thirdly, the specificity was evaluated using other allergens in soybeans. As shown in Figure 5, adding 20 μg mL^−1^ of β-conglycinin, trypsin inhibitor, and lectin to wells did not significantly decrease the fluorescent intensities of QB-pAbs. Only when 0.62 μg mL^−1^ of glycinin was added did the fluorescent intensities of QB-pAbs decrease significantly, which shows that the QB-pAbs probes could avoid the interference from other soybean allergens.

### 2.5. Comparison of QBs-FLISA and Common ELISA

To further assess the analytical performance of the QBs-FLISA, we compared QBs-FLISA with conventional ELISA using the same coated antigens and pAbs. The linear range of the calibration curve of ELISA ranged from 0.5 to 32 μg mL^−1^ (R^2^ = 0.99). Critical parameters of ELISA and QBs-FLISA were summarized in Table 3. Compared to the ELISA, QBs-FLISA demonstrated simplified detection processes, a lower detection limit, and reduced detection time. For example, compared with ELISA, the detection sensitivity of glycinin using QBs-FLISA was 7-fold increased times (shown as LOD), which showed that the FLISA has significant commercial potential. In addition, several soybean samples were detected, and the data indicated that there was no significant difference in detection results between the two methods (Table 4). Therefore, QBs-FLISA exhibited significant commercial potential, especially for these soybean samples containing lower glycinin.

### 2.6. Analysis of Real Samples

**Figure 6:**
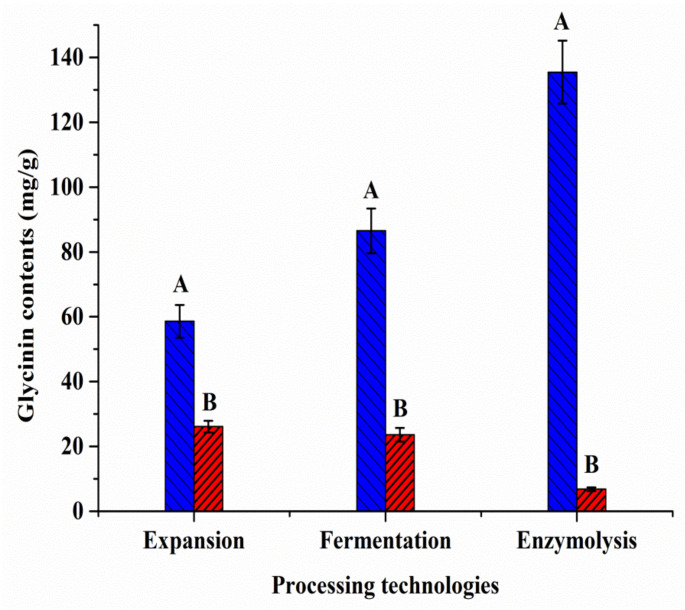
The glycinin contents of soybean products before (A columns) and after (B columns) different processing methods.

Several processing techniques have been developed in recent years to decrease the activity of soybean allergens. The method established in this study can also be used to accurately evaluate the effect of various processing procedures on the amount of glycinin in soybean products. Here, QBs-FLISA was employed to evaluate three typical soybean processing technologies (including expansion, fermentation, and enzymolysis). The contents of glycinin in extruded full-fat soybean, fermented soybean meal, and proteolytic soybean meal were 26.15, 23.66, and 6.85 mg/g, respectively, indicating a reduction of about 55.39%, 72.67%, and 94.94% compared with the same soybean samples before processing (Figure 6). These results indicate that the three processes were capable of effectively removing glycinin from soy products. In particular, glycinin was almost eliminated using enzymatic hydrolysis.

In addition, we randomly collected 4 kinds of soybean processing products from major soybean processing enterprises in China. As shown in Table 5, the average content of glycinin in soybean meal, extruded soybean, fermented soybean meal, and proteolytic soybean meal were 105.3 ± 23.7 mg/g, 55.1 ± 18.9 mg/g, 45.1 ± 31.0 mg/g, and 11.2 ± 14.8 mg/g, respectively. The results show that the effects of the four soybean processing methods were extraction < expansion < fermentation < enzymolysis. That is, enzymatic hydrolysis is the best way to reduce glycinin. Due to the thermal stability of glycinin, high temperature, high pressure, and shear force used in the extrusion of soybean meal have limited the ability to destroy glycinin [25]. Glycinin content in soybean meal was the highest. Because of the parameters for extruded full-fat soybean, including heating temperature, heating time, and moisture content, were more stringent than those for extruded soybean meal [7], the effect of expansion was better than that of extraction, but there was still an average of 55.1 ± 18.9 mg/g glycinin detected in extruded full-fat soybean samples. Microbial fermentation has previously been demonstrated to breakdown 78.28% of glycinin in soybean meal into small-size peptides or amino acids [26]. However, our test results show that the content of glycinin in fermented soybean meals varied greatly from different enterprises. Some studies have shown that the difference of soybean meal fermentation may be related to the differences of microorganisms, fermentation substrate, fermentation temperature, and other process parameters in the fermentation process [27,28]. Additionally, as a frequently used form of protein modification, enzymatic hydrolysis may eliminate allergen epitopes or break chemical bonds in soybean antigen protein. Our results also indicate that enzymatic hydrolysis was the most effective process to reduce glycinin, and the glycinin content was greatly reduced during enzymolysis, with certain enzymolysis procedures even totally removing glycinin.

## 3. Materials and Methods

### 3.1. Reagents and Instruments

CdSe/ZnS QB (Ex/Em: 305/630 nm) were obtained from Shanghai Kundao Biotechnology Co., Ltd (Shanghai, China). Goat anti-rabbit immunoglobulin horseradish peroxidase (IgG-HRP) was purchased from JiSen Biotechnology Co., Ltd (Yulin, China). Freund’s complete and incomplete adjuvants, 1-ethyl-3-(3-dimethylaminopropyl) carbodiimide (EDC), o-phenylenediamine were purchased from Sigma-Aldrich (St. Louis, MO, USA). PierceTM Protein A sepharose column was purchased from Thermo Fisher Scientific (Waltham, MA, USA). Tween 20 and bovine serum albumin (BSA) were purchased from Solarbio Technology Co., Ltd(Beijing, China). Other reagents were analytically pure, and water was purified using a Milli-Q system (Millipore, Bedford, MA, USA) (resistivity, 18.2 MΩ·cm). Ninety-six-well microplates were purchased from Costar (Corning, New York, NY, USA).

Size exclusion chromatography separation was performed on an Agilent 1200 HPLC system coupled with a diode array detector (Agilent Technologies, Fremont, CA, USA). The hydrodynamic size of QB and conjugated QB-pAbs were analyzed by a dynamic light scattering particle size analyzer (Malvern, Nano ZS). The images of CdSe/ZnS QBs were taken with JEM 1230 transmission electron microscopes (TEM) under 80 kV. X-ray photoelectron spectroscopy (XPS) measurements were conducted using an ESCALAB 250Xi spectrometer (ThermoFisherVG Scientific, Waltham, MA, USA) with monochromatized Al Kα X-rays (energy = 1486.6 eV). Ninety-six-well plates were read by BioTek Synergy4 microplate (Gene Company Limited, Hong Kong, China).

### 3.2. Preparation and Purification of Glycinin

The raw soybean seeds were crushed to achieve a particle size of 60 um. Defatting and extracting the soybeans powder with n-hexane and 0.03 mol/L of Tris-HCl buffer (containing 0.01 mol L^−1^ β-mercaptoethanol, pH 8.0) (1:15 solid-liquid ratio) for 2 h shaking (1200 rpm) at room temperature, then the mixtures were centrifuged for 20 min (8000 rpm). The obtained supernatant was treated with 2 mol/L of HCl to adjust pH to 6.4 and maintained at 4°C overnight. After centrifugation for 20 min at 4 °C (8000 rpm), the precipitate was crude soybean glycinin. The extract was further purified by a Waters XBridge^®^ BEH SEC 200 Å column (3.5 μm particle size, 7.8 × 300 mm) in the HPLC system. The column temperature was room temperature, the injection volume was 50 μL, the mobile phase was 50 mM pH 7.5 sodium phosphate buffer solution, and the flow rate was 0.5 mL/min. The detection wavelength of diode array detector was set to 280 nm, and the peak solution of 10–12 min was collected. Then, the purity of purified glycinin was analyzed using SDS-PAGE and HPLC, and the concentration was determined by BCA TM protein kit.

### 3.3. Production of Polyclonal Antibodies

Antibodies were produced using New Zealand white rabbits. Crude soybean glycinin in a quantity of 0.5 mg emulsified in complete Freund’s adjuvant was injected subcutaneously in each rabbit, and four weeks after the first immunization, three more booster injections were administered at two-week intervals for a total of three with half the antibody dose and emulsified in Freund’s incomplete adjuvant. Ten days following the last injection, blood samples from the heart were collected and centrifuged for 20 min at 4000 rpm. The antibodies from the antiserum were purified by a protein A-sepharose column. Characterization of purified glycinin and antibodies are included in the Supplementary Materials.

### 3.4. Synthesis of QB-pAbs Probes

QB-pAbs conjugates were prepared by covalently reacting carboxyl groups on the nanobeads with amino groups on the antibodies according to previously reported procedures [24]. Briefly, 30 μL of 10 mg mL^−1^ QB was dispersed in pH 6.0 phosphate buffer (PB) and reacted with 20 mg mL^−1^ EDC (3 μL). After oscillating for 25 min at 37 °C, the mixture was centrifugated at 14,800 rpm. Discarding the supernatant, precipitates (QB) were redissolved in PB (130 μL, pH 7.4). Then, glycinin pAbs were added into QB solution to incubate for 30 min at 37 °C. Then, the QB-glycinin pAbs were blocked using 20 mg mL^−1^ BSA solution and stored in 100 μL of 0.05 M Tris-HCl containing 0.2% PC300, 0.9% NaCl, 5% BSA, and 10% trehalose.

### 3.5. Fabrication of QBs-FLISA

Firstly, 5 μg mL^−1^ purified glycinin was dissolved with NaHCO_3_/Na_2_CO_3_ buffer (pH 9.6) and added into the wells (100 μL per well). After incubation at 4 °C overnight, the redundant binding sites were blocked with 200 μL of NaHCO_3_/Na_2_CO_3_ buffer containing 1% BSA for 1 h at 37 °C, then the wells were washed three times with PBST (0.01 M PBS with 0.1% Tween 20, pH 7.0). Subsequently, 50 μL of different concentrations of glycinin sample extracts (or standard solutions) and 50 μL of QB-pAbs (40-times diluting with 0.01 M PBS, pH 7.0) were mixed in wells and incubated at 37 °C for 45 min. After washing with PBST three times, 200 μL of PBS buffer was added to each well on plates and measured using a microplate reader at a wavelength of 305 nm excitation and 630 nm emission.

### 3.6. Procedures of Common ELISA

The ELISA plates were coated with 10 μg mL^−1^ purified glycinin dissolved in NaHCO_3_/Na_2_CO_3_ buffer (100 μL per well), and allowed to incubate at 4 °C overnight. After blocking using BSA, anti-glycinin pAbs were added (50 μL per well) in the presence of 50 μL per well different concentrations of glycinin standard solutions (or sample extracts) and incubated at 37 °C for 1 h. Goat anti-rabbit IgG-HRP conjugate was added and incubated for 1 h. An o-phenylenediamine substrate solution was applied to color development for 15 min, 2 M H_2_SO_4_ (50 μL per well) was added to stop the color development. The plates were read at 492 nm with a reference wavelength of 570 nm.

### 3.7. Sample Pretreatment

The samples were crushed to achieve a particle size of 60 um, and 0.03 g samples were weighed into a 50 mL centrifuge tube. Soybean powder was extracted using 20 mL extraction buffer solutions (50 mM Tris-HCl including 0.05 mol L^−1^ β-mercaptoethanol) and shaken with vortex oscillation (260 rpm) at 37 °C for 1 h. The supernatant was centrifuged for 5 min at 4000 rpm. Then, the supernatant was diluted to within the concentration range of the glycinin standard curve with 0.01 M PBS (pH 7.0).

### 3.8. Method Validation

Method validity, including linearity, sensitivity, accuracy, and precision were evaluated. The limit of detection (LOD) was defined as the glycinin standard solution concentration that prevented 10% of QB-pAbs to bind with glycinin coated in microplate [22]. It was determined as the glycinin concentration when F/F0 = 0.9, in which F and F0 referred to the fluorescent intensity of wells after incubated with positive sample solutions and negative sample solutions (0.01 M PBS, pH 7.0), respectively. Accuracy evaluation was performed by detecting soybean, extruded soybean, fermented soybean meal, and proteolytic soybean meal samples. These samples were spiked with purified glycinin solution at 40, 80, and 120 mg g^−1^. The relative standard deviation (RSD) of 6 replications was used to assess precision.

### 3.9. Analysis of Glycinin in Real Soybean Seeds and Soy Products

In 2020, 10 distinct commercial types of soybean seeds were harvested from various growing locations (Heilongjiang, Hubei, Shandong, and Hunan provinces, China). Ten distinct products derived from each of the 4 processing procedures (extraction, expansion, fermentation, and enzymolysis) were gathered from Chinese major soybean processing firms. All samples were crushed into powder and mixed homogeneously and were then stored at −80 °C until detection. The method assessment, characterization, and validation studies used a total of five raw soybeans and soy products.

### 3.10. The Data Analysis

The FLISA and ELISA data were analyzed using OriginPro 9.0 software. A calibration curve was constructed using serial dilutions of purified glycinin as standard, and semi log coordinate was used for linear regression analysis.

## 4. Conclusions

In summary, after isoelectric point crude extraction and SEC chromatographic column purification, soybean glycinin with a purity of more than 90% was obtained, which provided a good quantitative reference for the accurate determination of glycinin. Based on that, a novel competitive FLISA platform for the determination of glycinin was successfully developed with QB-labeled polyclonal antibodies serving as fluorescent probes. QB emitted a bright fluorescent signal contributing to significantly increase the detection sensitivity. The LOD reached 0.35 μg mL^−1^ for glycinin. The QBs-FLISA demonstrated high sensitivity and simplified analytical process in comparison to ELISA. In addition to monitoring glycinin in soybean and soy products, the method can be extended to detect other allergens in feed and food.

## Figures and Tables

**Table 1:** The optimization of coated glycinin concentrations and QB-pAbs dilution ratios using checkerboard (*n* = 3).

Coated Glycinin Concentration (μg/mL)	Dilution Ratios of QB-pAbs		
	10 Times	20 Times	40 Times
20	2476	1380	581
5	2095	1180	586
2.5	1344	1078	467
1	619	536	274
0.5	452	323	217
0.25	215	160	194
0	206	193	63

**Table 2:** Recoveries of glycinin spiked in various soybean products (*n* = 6).

Soybean Products	Spiked Level (mg/g)	Measured Concentration (mg/g)	Recovery (%)	CV (%)
Soybean	40	37.0	92.5	7.3
	80	77.3	96.7	2.4
	120	124.08	103.4	5.7
Extruded soybean	40	35.92	89.8	8.1
	80	77.6	97.0	6.3
	120	114.48	95.4	5.6
Fermented soybean meal	40	36.32	90.8	8.6
	80	84.32	105.4	4.3
	120	112.32	93.6	7.7
Proteolytic soybean meal	40	42.26	105.6	8.6
	80	76.16	95.2	6.0
	120	120.16	100.1	7.2

**Table 3:** Comparison of the developed QBs-FLISA and ELISA.

Method	LOD (μg mL^−1^)	Linear Range (μg mL^−1^)	Detection Time (min)
ELISA	0.25	0.5–32 μg/mL	135
QBs-FLISA	0.035	0.075–5 μg/mL	45

**Table 4:** The QBs-FLISA and ELISA results of different soybean products (*n* = 6).

Soybean Products	Glycinin Concentration (mg/g)		Relative Deviation (%)
	QBS-FLISA	ELISA	
Soybean	46.2	45.1	1.2
Soybean meal	126.5	129.3	1.1
Extruded soybean	19.9	21.2	3.2
Fermented soybean meal	24.3	23.7	1.2
Proteolytic soybean meal	7.5	6.8	4.9

**Table 5:** Concentrations of glycinin in soybean samples with different processings.

No.	Soybean Products	Glycinin Concentration (mg/g)	No.	Soybean Products	Glycinin Concentration (mg/g)
1	Soybean seed	85.4	31	Extruded soybean	42.52
2		71.0	32		70.2
3		82.2	33		82.1
4		85.7	34		48.9
5		66.1	35		50.2
6		67.1	36		48.8
7		103.8	37		53.6
8		112.1	38		23.6
9		108.3	39		85.4
10		106.2	40		45.2
11		132.1	41	Fermented soybean meal	34.1
12		88.9	42		4.3
13		59.7	43		63.1
14		90.6	44		9.6
15		78.3	45		7.3
16		69.4	46		73.5
17		109.8	47		86.3
18		113.4	48		75.9
19		76.4	49		62.0
20		88.6	50		35.4
21	Soybean meal	83.1	51	Proteolytic soybean meal	21.7
22		92.0	52		2.9
23		124.8	53		1.6
24		128.9	54		17.1
25		73.5	55		4.3
26		134.02	56		47.8
27		83.18	57		12.1
28		86.10	58		1.2
29		129.34	59		0.1
30		118.26	60		3.6

## Data Availability

Not applicable.

## Supplementary Materials

The following supporting information can be downloaded at: https://www.mdpi.com/article/10.3390/molecules27123664/s1, Figure S1: Purification of glycinin and anti-glycinin polyclonal antibody determined by SDS-PAGE. From left to right: Line 1, protein molecular weight marker(kDa); Line 2, Purified antibody; Line 3, rabbit serum; Line 4, soybean extract; Line 5, the purified glycinin.; Figure S2: The high performance liquid chromatograms of (A) the soybean extact and (B) purified glycinin.; Table S1: OD450 values of wells with different anti-glycinin pAbs dilutions.

## Sample Availability

Samples of the compounds are not available from the authors.
