# Supplementary material for: Quantum Dot Nanobead-Based Fluorescence-Linked Immunosorbent Assay for Detection of Glycinin in Soybeans and Soy Products
Source: Molecules. 2022 Jun 7;27(12):3664. doi: 10.3390/molecules27123664 (PMC9228775; doi:10.3390/molecules27123664)
Supplement: Supplementary file 1 [file molecules-27-03664-s001.zip › molecules-1741984-supplementary.pdf]

Quantum dot nanobeads-based fluorescence-linked immunosorbent assay for detection of glycinin in soybeans and soy products

Qinglong Song†, Anguo Liu†, Shimin Zhang, Runxian Li, Shiyan Qiao, Pingli He*

State Key Laboratory of Animal Nutrition, College of Animal Science and Technology, China Agricultural University, Beijing 100193, China; sql19972002@126.com (S.Q.); angelo524@foxmail.com (A.L.); 1109446827@qq.com (S.Z.); lrx1024@cau.edu.cn(R.L.); qiaoshiyan@cau.edu.cn (S.Q.)

* Correspondence: hepingli@cau.edu.cn; Tel.: + 86-10-62733588

† These authors contributed equally to this work.

Characterization of purified glycinin and antibodies

Size exclusion chromatography (SEC) is a chromatographic technique used to separate proteins by their size in solution, which could significantly increase resolution compared to currently available separation columns, such as SE-HPLC. In this study, glycinin was extracted with Tris-HCl and precipitated at isoelectric point, and further purified with size exclusion chromatography (SEC) by HPLC. The SDS-PAGE and HPLC analysis results show that the purity of soybean glycinin is greater than 90% (Figure S1 & Figure S2). The preparation of high purity glycinin provides a good quan-titative reference for accurate determination of its content in soybean and soy products. Meanwhile, after purification using protein A sepharose column according to the recommended procedure, the pAbs had high purity with a titer of more than 1:50000 (Figure S1 & Table S1).

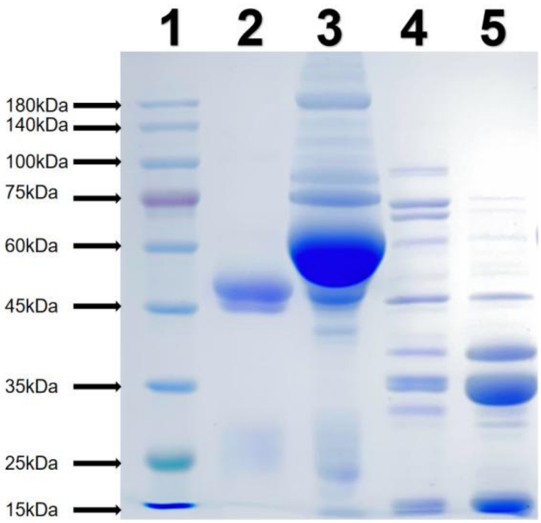

Figure S1. Purification of glycinin and anti-glycinin polyclonal antibody determined by SDS-PAGE. From left to right: Line 1, protein molecular weight

marker(kDa); Line 2, Purified antibody; Line 3, rabbit serum; Line 4, soybean extract; Line 5, the purified glycinin.

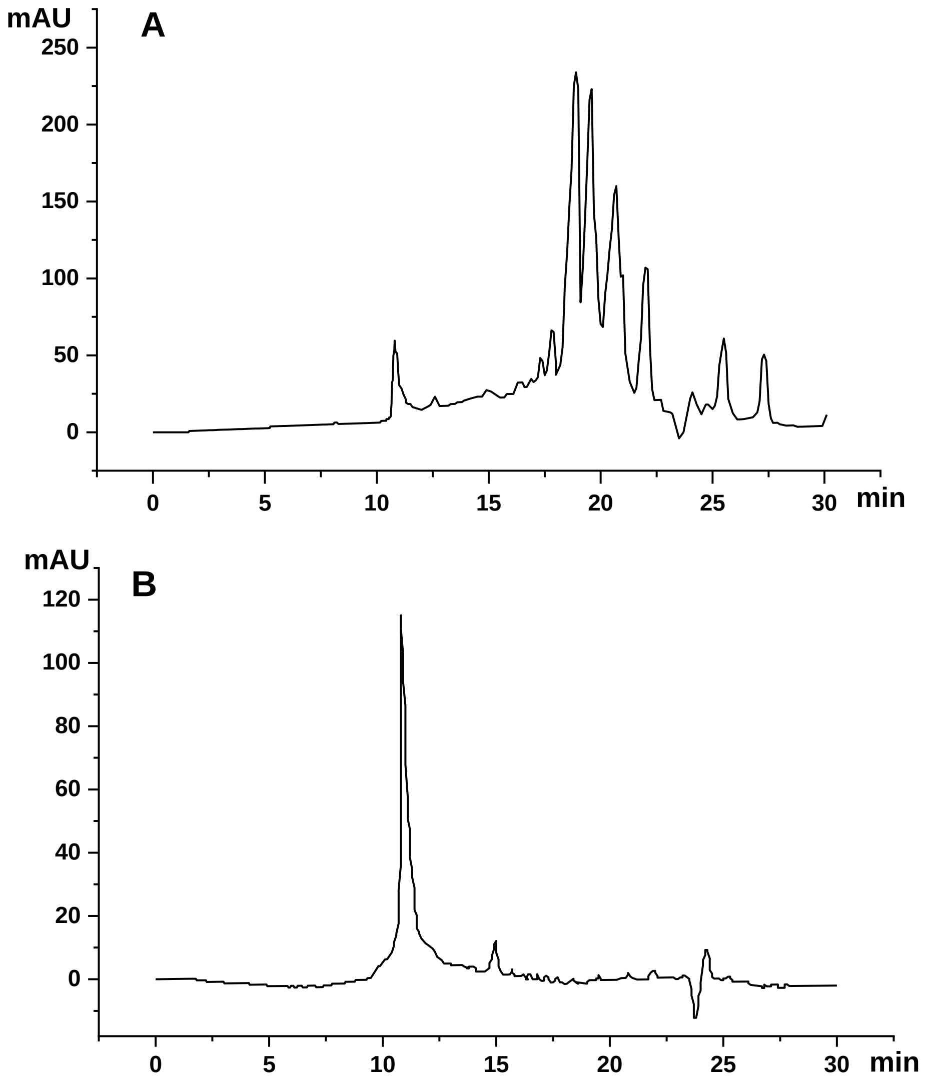

Figure S2. The high performance liquid chromatograms of (A) the soybean extact and (B) purified glycinin.

Table S1. OD450 values of wells with different anti-glycinin pAbs dilutions.

Item	Dilution of anti-glycinin serum (Times)					
	500	1,000	5,000	10,000	50,000	Negative control
OD_{450} value	2. 891	2.487	1.312	0.325	0.092	0.020
